# Six-Month Cardiometabolic and Vascular Trajectories After COVID-19 Hospitalization: Exploratory Associations with Recorded Dietary and Omega-3 Management

**DOI:** 10.3390/jcm15145647

**Published:** 2026-07-18

**Authors:** Cristiana Adina Avram, Maria Rada, Ana-Maria Pah, Gheorghe Stoichescu-Hogea, Diana-Maria Mateescu, Ioana Cotet, Claudia Raluca Balasa Virzob, Dan Alexandru Surducan, Abdeldayem Emad Mahmoud, Claudiu Avram, Maria-Laura Craciun

**Affiliations:** 1 Department of Internal Medicine I, “Victor Babeș” University of Medicine and Pharmacy of Timișoara, Eftimie Murgu Square 2, 300041 Timișoara, Romania; avram.adina@umft.ro; 2 Department of Cardiology, “Victor Babeș” University of Medicine and Pharmacy of Timișoara, Eftimie Murgu Square 2, 300041 Timișoara, Romania; rada.maria@umft.ro (M.R.); laura.craciun@umft.ro (M.-L.C.); 3 Department of Infectious Diseases, Clinical Hospital CF Timișoara, 1 Gării Street, 300166 Timișoara, Romania; diana.mateescu@umft.ro; 4 Doctoral School, Faculty of Medicine, “Victor Babeș” University of Medicine and Pharmacy of Timișoara, Eftimie Murgu Square 2, 300041 Timișoara, Romania; ioana.cotet@umft.ro; 5 Department of Clinical Nursing, “Victor Babeș” University of Medicine and Pharmacy of Timișoara, Eftimie Murgu Square 2, 300041 Timișoara, Romania; virzob.claudia@umft.ro; 6 Department of Functional Sciences, Discipline of Public Health, Center for Translational Research and Systems Medicine, “Victor Babeș” University of Medicine and Pharmacy of Timișoara, Eftimie Murgu Square 2, 300041 Timișoara, Romania; surducan.dan@umft.ro; 7 Centre for Translational Research and Systems Medicine, Faculty of Medicine, “Victor Babeș” University of Medicine and Pharmacy of Timișoara, Eftimie Murgu Square 2, 300041 Timișoara, Romania; 8 Department of Family Medicine, “Victor Babeș” University of Medicine and Pharmacy of Timișoara, Eftimie Murgu Square 2, 300041 Timișoara, Romania; mahmoodemadsalama@gmail.com; 9 Department XVI—Balneology, Medical Rehabilitation and Rheumatology, “Victor Babeș” University of Medicine and Pharmacy of Timișoara, Eftimie Murgu Square 2, 300041 Timișoara, Romania; avram.claudiu@umft.ro; 10 Transdisciplinary Research Center for Medical Rehabilitation, Balneology and Rheumatology (CTRMBR), “Victor Babeș” University of Medicine and Pharmacy of Timișoara, Eftimie Murgu Square 2, 300041 Timișoara, Romania

**Keywords:** COVID-19, post-COVID follow-up, cardiometabolic risk, dietary counseling, omega-3 fatty acids, longitudinal cohort, observational study, vascular markers

## Abstract

**Background/Objectives**: Post-COVID follow-up may provide an opportunity to reassess persistent and modifiable cardiometabolic risk. We characterized six-month cardiometabolic and vascular trajectories after COVID-19 hospitalization and explored associations with routinely documented dietary counseling and omega-3 recommendations. **Methods**: This single-center retrospective longitudinal cohort included 238 adults hospitalized with COVID-19 between March 2020 and December 2024. Patients were classified according to recorded post-discharge management as usual care (*n* = 64), dietary management (*n* = 60), omega-3 supplementation (*n* = 59), or combined management (*n* = 55). Outcomes were assessed at the index visit and at visits labeled approximately three and six months later. Longitudinal changes were estimated using generalized estimating equations; six-month associations were evaluated using baseline-adjusted ANCOVA with robust standard errors and Benjamini–Hochberg correction. **Results**: Mean age was 54.0 ± 10.6 years, 54.6% were women, 82.4% had hypertension, 40.8% had diabetes, and mean body mass index was 33.8 ± 4.6 kg/m^2^. At six months, endpoint-specific data were available for 182 participants for blood pressure, total cholesterol, triglycerides, and HDL-C; 180 for waist circumference; 179 for SCORE; 177 for LDL-C and bilateral carotid IMT analyses; 173 for HOMA-IR; and 164 for IL-6. Across the cohort, systolic and diastolic blood pressure decreased by 7.91 and 4.75 mmHg; several lipid, anthropometric, and insulin-resistance markers also improved. No omnibus group-by-time interaction was significant. After false-discovery-rate correction, only higher six-month HDL-C in the dietary and combined-management groups versus usual care remained statistically significant; all other between-group findings were nonsignificant after correction. **Conclusions**: Several cardiometabolic risk markers improved during follow-up, but no recorded management category showed consistent trajectory-level superiority. Because intervention content and adherence, time-updated medication use, acute COVID-19 severity, and lifestyle changes were not captured, the comparative findings are exploratory and noncausal. The isolated HDL-C associations should not be interpreted as evidence of cardiovascular benefit. No cardiac biomarkers, echocardiographic measures, incident heart failure, or cardiovascular events were assessed.

## 1. Introduction

COVID-19 is increasingly recognized as a systemic disease with cardiovascular consequences that may extend beyond the acute infection. Large-scale longitudinal analyses have shown an excess post-acute burden of dysrhythmias, ischemic and nonischemic heart disease, thromboembolic disease, and heart failure (HF), with risk increasing according to the severity of the index illness [1]. Hospitalization with COVID-19 has also been associated with a higher subsequent risk of incident HF, particularly among older and hypertensive survivors [2,3].

The mechanisms linking severe acute infection to later cardiometabolic risk are likely multifactorial and may include myocardial injury, endothelial dysfunction, autonomic disturbance, persistent inflammation, deconditioning, renal dysfunction, insulin resistance, obesity, and destabilization of pre-existing cardiovascular disease [4]. These observations support systematic reassessment of modifiable cardiovascular risk after hospitalization. Although blood-pressure, glycemic, lipid, and weight control are relevant to primary cardiovascular prevention and may also influence future HF risk [5,6], such risk-factor management should not be equated with demonstration of HF prevention in the absence of cardiac biomarkers, imaging, or clinical HF outcomes.

Post-COVID care pathways have largely focused on persistent symptoms and exclusion of overt myocardial involvement. Expert guidance recommends symptom-directed cardiovascular evaluation and graded return to physical activity, but evidence for scalable cardiometabolic follow-up pathways remains limited [4]. Dietary quality, weight management, blood-pressure control, and lipid optimization are established components of preventive cardiology [7,8]. Marine omega-3 fatty acids may lower triglycerides and modestly reduce blood pressure, although their effects depend on dose, formulation, clinical context, background therapy, and adherence [9,10,11]. Whether routinely documented dietary counseling or omega-3 recommendations are associated with differential cardiometabolic recovery after COVID-19 hospitalization remains uncertain.

The present study evaluated adults hospitalized with COVID-19 and followed for six months. The objectives were: (i) to quantify population-average changes in hemodynamic, glycometabolic, lipid, inflammatory, vascular, and estimated cardiovascular-risk markers; (ii) to explore associations between recorded dietary and omega-3 management categories and six-month outcomes after adjustment for measured baseline differences; and (iii) to define the limitations of interpreting these routinely recorded exposures as interventions. The study contributes a multidomain longitudinal description of post-hospitalization cardiometabolic trajectories and a cautious real-world comparison of nonstandardized management categories. Because allocation was nonrandomized, important treatment-selection variables were unavailable, and no HF-specific outcomes were measured, all comparative analyses were prespecified as exploratory and noncausal.

## 2. Materials and Methods

### 2.1. Study Design, Setting, and Participants

This was a single-center retrospective longitudinal observational cohort study conducted at the Clinical Hospital of Infectious Diseases and Pneumophysiology, Dr. Victor Babeș Timișoara, Romania. The analytic cohort comprised 238 adults hospitalized with COVID-19 between March 2020 and December 2024. COVID-19 was documented by reverse transcription-polymerase chain reaction testing of nasopharyngeal swabs according to institutional protocols and the national guidance applicable during the study period. The index cardiometabolic assessment occurred at hospital discharge or at the first post-discharge outpatient cardiometabolic visit within 30 days after discharge. Subsequent records were categorized by the source database as approximately three-month and six-month visits. Exact visit dates and day-level intervals were not consistently retained; therefore, medians, ranges, and visit-window distributions could not be reconstructed. Eligible records were identified from the hospital electronic medical record and information system, and all consecutive records meeting the prespecified eligibility criteria were included; no random or convenience sampling was applied. The analytical extract began with the final eligible cohort and did not retain the total number of records initially queried or the number excluded at each screening step.

Eligible participants were adults with laboratory-confirmed COVID-19, an analyzable index cardiometabolic record, a documented post-discharge management category, and written informed consent for use of de-identified clinical data. Adults were not excluded solely because of pre-existing cardiovascular disease, and patients with and without hypertension, diabetes, or dyslipidemia were included. Pre-existing cardiovascular diagnoses other than these recorded risk factors were not uniformly adjudicated, and the database did not contain sufficiently complete information on prior HF, natriuretic peptides, left-ventricular ejection fraction, cardiac structure, or filling pressures to assign a formal HF stage. Standardized measures of acute COVID-19 severity were also unavailable. No a priori sample-size or power calculation was performed because all eligible records in the source database were included. Consequently, the study may have had limited power to detect modest between-group differences, and nonsignificant estimates were interpreted using their confidence intervals rather than as evidence of equivalence. Patients were excluded if they had no analyzable index cardiometabolic record, no documented management category, or no written informed consent. Prior use of dietary supplements, including omega-3 preparations, was not systematically documented and could not be applied as an exclusion criterion.

### 2.2. Real-World Management-Strategy Classification

At the index assessment, patients were classified according to the post-discharge management category documented in the electronic medical record: usual care, dietary management, omega-3 supplementation, or combined dietary management plus omega-3 supplementation. These categories represent recorded clinical exposures, not protocolized study interventions. Dietary management was defined as documented dietary counseling or a structured recommendation, typically Mediterranean-style or hypocaloric, emphasizing vegetables, fruit, whole grains, legumes, nuts, fish, and unsaturated fats while limiting sodium, refined carbohydrates, and ultra-processed foods. Counseling was generally delivered individually by the treating physician or, when available, a clinical nutritionist, usually at or shortly after discharge and occasionally reinforced at follow-up. The number, frequency, duration, and content of contacts were not recorded uniformly. Omega-3 management was defined as a documented prescription or recommendation for a commercially available marine fish-oil preparation containing EPA and DHA. Product, EPA/DHA composition, dose, initiation date, intended duration, continuation, and adherence were not consistently captured. Adherence was not assessed by questionnaire, pill count, pharmacy refill, or biomarker. Management category was abstracted from discharge summaries and outpatient notes containing free-text or checkbox entries; no dedicated case-report form, food-frequency questionnaire, or supplement log was used. This retrospective exposure ascertainment introduces potential information bias and within-category heterogeneity, and precludes reproducible dose–response or per-protocol interpretation.

Strategy assignment was nonrandomized and reflected physician judgment, comorbidity profile, access, and patient preference. All estimates were therefore interpreted as adjusted associations rather than causal treatment effects. Broad cardiovascular medication classes were recorded at baseline, including beta-blockers, angiotensin-converting enzyme inhibitors or angiotensin-receptor blockers, calcium-channel blockers, diuretics, statins, and antiplatelet agents. Time-updated initiation, discontinuation, or dose intensification was not systematically captured. Detailed exposure to ezetimibe, PCSK9 inhibitors, GLP-1 receptor agonists, SGLT2 inhibitors, anti-obesity pharmacotherapy, and other contemporary cardiometabolic treatments was also unavailable. These therapies, together with changes in antihypertensive treatment, could substantially influence blood pressure, lipid profile, insulin resistance, body weight, and waist circumference. Consequently, neither population-average improvement nor between-category differences can be attributed to dietary counseling or omega-3 recommendations alone.

### 2.3. Clinical, Laboratory, and Vascular Measurements

Clinical variables included age, sex, smoking status, documented hypertension and diabetes mellitus, systolic blood pressure (SBP), diastolic blood pressure (DBP), body weight, body mass index (BMI), waist circumference, body-fat indices, and cardiovascular medication use. Anthropometric measurements were recorded using calibrated institutional equipment: height with a wall-mounted stadiometer, weight with a digital scale while participants wore light indoor clothing and no shoes, and waist circumference at the end of normal expiration at the midpoint between the lowest palpable rib and the superior border of the iliac crest. Blood pressure was measured by trained clinical staff using validated automated oscillometric devices with an appropriately sized cuff, with the participant seated, the arm supported at heart level, and at least five minutes of rest. The source records did not consistently retain the number of readings at each visit. When repeated readings were documented, their mean was used; when only one reading was recorded, that value was analyzed. Uniform use of duplicate or triplicate measurements could not be verified retrospectively.

All laboratory analyses were performed in the hospital’s accredited central laboratory using automated analyzers. Fasting plasma glucose, total cholesterol, triglycerides, HDL cholesterol, and LDL cholesterol were measured by enzymatic colorimetric methods on a Roche Cobas c501 chemistry analyzer (Roche Diagnostics, Mannheim, Germany). Insulin and interleukin-6 (IL-6) were measured by electrochemiluminescence immunoassay on a Roche Cobas e411 analyzer. The lower limit of detection for IL-6 was 1.5 pg/mL, with an intra-assay coefficient of variation <5% and an inter-assay coefficient of variation <8%. IL-6 was selected because it is a central mediator of the host cytokine response to SARS-CoV-2, has been associated with COVID-19 severity and survival, and was the inflammatory analyte most consistently available in the source database [12]. Broader cytokine and high-sensitivity C-reactive protein panels were not uniformly recorded. HOMA-IR was calculated as fasting insulin (µU/mL) × fasting plasma glucose (mg/dL)/405 [13]. Glucose, insulin, and lipid samples were obtained after an overnight fast of at least eight hours at the index and follow-up assessments; nonfasting samples were not used.

Ten-year fatal cardiovascular risk was summarized using the legacy SCORE value already stored in the source database [14]. The score was retained solely as an exploratory repeated marker because it was the only cardiovascular-risk estimate recorded consistently across the study period and was not retrospectively recalculated. SCORE2 was not substituted because it was developed for individuals without diabetes, while 40.8% of this cohort had diabetes [15]. SCORE2-Diabetes could not be reconstructed consistently because age at diabetes diagnosis, HbA1c, estimated glomerular filtration rate, and other required inputs were incomplete at repeated visits [16]. SCORE should therefore not be interpreted as a contemporary individualized risk classification. Carotid intima-media thickness (IMT) was measured separately on the left and right far walls of the common carotid artery, 1 cm proximal to the carotid bulb, using a 7–12 MHz linear-array transducer on an Aixplorer MACH 30 ultrasound system (SuperSonic Imagine S.A., Aix-en-Provence, France). The mean of three measurements was recorded on each side by experienced sonographers following the Mannheim consensus recommendations [17]. Formal study-specific intraobserver and interobserver reproducibility coefficients were not available in the source records.

### 2.4. Outcomes

Analyzed outcomes were SBP, DBP, HOMA-IR, total cholesterol, triglycerides, HDL-C, LDL-C, waist circumference, IL-6, legacy SCORE, and bilateral carotid IMT. Because no outcome hierarchy or statistical analysis plan was prospectively registered and management allocation was nonrandomized, all longitudinal and between-category analyses were considered exploratory. The study did not designate a confirmatory primary treatment endpoint.

The study did not assess natriuretic peptides, echocardiographic structure or function, filling pressures, incident HF, HF hospitalization, cardiovascular events, cardiovascular mortality, exercise capacity, or validated patient-reported outcomes. It therefore evaluates cardiometabolic and vascular risk markers rather than demonstrating prevention of HF or HF with preserved ejection fraction.

### 2.5. Data Quality and Missing Data

The analytical dataset was de-identified before analysis. Range and consistency checks were applied to numerical fields. Two follow-up BMI values (2.5 and 2.7 kg/m^2^) were excluded as implausible data-entry errors; no baseline BMI values used for covariate adjustment were excluded. Outcome-specific denominators were reported because biomarker and follow-up availability differed. No values were imputed, and available-case analyses were used. Retrospective extraction from free-text and checkbox documentation could have produced exposure misclassification or incomplete capture of counseling, supplementation, medications, lifestyle changes, and follow-up timing.

Availability of core blood-pressure outcomes was 79.0% at the visit labeled three months and 76.5% at the visit labeled six months. Exact calendar intervals were unavailable. Because follow-up completeness may have depended on baseline risk, illness severity, treatment access, or engagement with care, missingness was not assumed to be completely at random, and attrition bias could not be excluded.

### 2.6. Statistical Analysis

Continuous variables are presented as mean ± standard deviation when approximately symmetric and as median [interquartile range] when skewed. Categorical variables are presented as number (percentage). Baseline comparisons used one-way analysis of variance, the Kruskal–Wallis test, or Pearson’s chi-squared test, as appropriate.

Population-average longitudinal changes were estimated using Gaussian generalized estimating equations with an exchangeable working correlation structure, patient-level clustering, and robust covariance estimates [18]. Time was modeled categorically, with the index assessment as the reference. Models included time, management group, age, sex, and baseline BMI; separate group-by-time interaction terms tested whether trajectories differed among strategies. HOMA-IR, triglycerides, and IL-6 were transformed as log(1 + value) because of right-skewed distributions. Back-transformed coefficients were expressed as approximate percentage differences.

Six-month between-category comparisons used analysis of covariance with the six-month value as the dependent variable and the corresponding baseline value, age, sex, baseline BMI, hypertension, diabetes, and current smoking as covariates. Blood-pressure models additionally included the number of baseline antihypertensive classes; lipid models included baseline statin use; and the HOMA-IR model included baseline diabetes treatment. Heteroscedasticity-consistent HC3 standard errors were used, and usual care was the reference category. Propensity-score matching and inverse-probability weighting were considered but were not implemented. The four-category exposure, modest group sizes, marked baseline imbalance, incomplete determinants of treatment selection, and inability to verify adequate overlap would have risked loss of precision or unstable weights. More importantly, propensity methods balance only measured variables and would not address the central unmeasured confounders in this dataset, including acute severity, vaccination, physical activity, socioeconomic factors, adherence, and medication changes [19]. Prespecified multivariable outcome regression was therefore retained, with explicit noncausal interpretation.

Two-sided *p* values < 0.05 were considered statistically significant. Because 36 category-specific comparisons were evaluated, the Benjamini–Hochberg procedure was applied to control the false discovery rate [20]. Both nominal *p* values and adjusted q values were reviewed, and only findings surviving false-discovery-rate correction were treated as statistically robust within this exploratory analysis. Pairwise standardized mean differences (SMDs) were calculated for continuous variables using the pooled standard deviation and for binary variables using the pooled binomial variance; Table 1 reports the maximum absolute pairwise SMD across the four groups. Post hoc standardized effect sizes for outcomes analyzed on their original scale were calculated by dividing adjusted estimates by the corresponding overall baseline standard deviation. No post hoc power calculation was performed, because such calculations do not resolve uncertainty after analysis; confidence intervals were used to assess the range of effects compatible with the data. Nonsignificant findings may reflect absence of a meaningful association or insufficient power, particularly for modest effects and outcomes with incomplete follow-up. Analyses were performed in Python 3.13.5 using NumPy 2.3.5, SciPy 1.17.0, and statsmodels 0.14.6. Reporting follows STROBE [21].

### 2.7. Ethics

The study was conducted in accordance with the Declaration of Helsinki and was approved by the Ethics Committee of the Clinical Hospital of Infectious Diseases and Pneumophysiology, Dr. Victor Babeș Timișoara (approval no. 11900, 16 December 2025). All participants provided written informed consent for participation and for use of their de-identified clinical data for research and scientific publication.

The clinical data analyzed were generated during routine medical care between March 2020 and December 2024. The ethics approval authorized the retrospective use, de-identification, analysis, and publication of these routine-care data. No research-specific intervention or prospective research data collection was undertaken as part of the present study.

## 3. Results

### 3.1. Cohort Characteristics and Follow-Up

**Figure 1:**
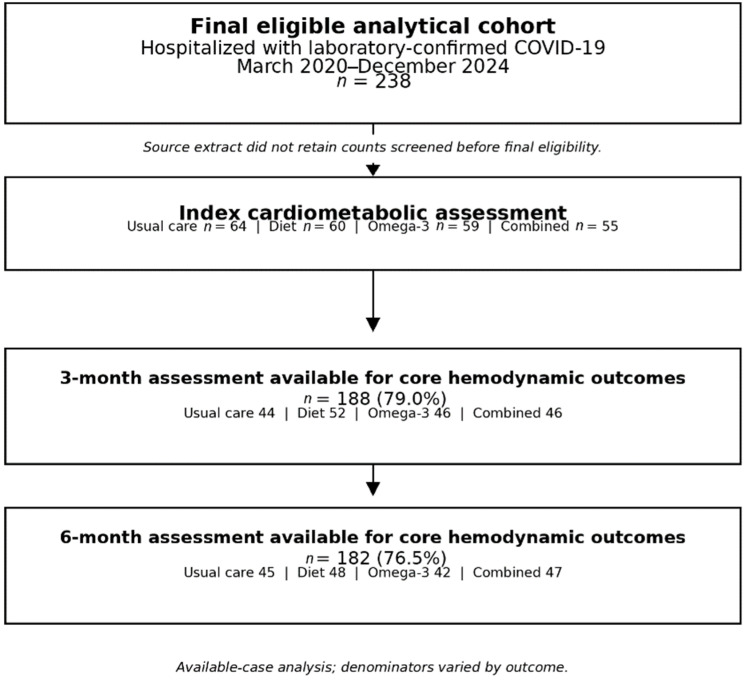
Final eligible analytical cohort and availability of core hemodynamic outcomes. The source extract did not retain the total number of records screened before final eligibility. Follow-up denominators varied by outcome; no outcome values were imputed.

The final eligible analytical cohort included 238 patients: 64 receiving usual care, 60 with recorded dietary management, 59 with recorded omega-3 supplementation, and 55 with combined management (Figure 1). The source extract did not permit reconstruction of the number of records screened before final eligibility. Mean age was 54.0 ± 10.6 years, 130 participants (54.6%) were women, 196 (82.4%) had hypertension, 97 (40.8%) had diabetes mellitus, and mean BMI was 33.75 ± 4.60 kg/m^2^. Current smoking was recorded in 47 patients (19.7%).

Baseline comparability was limited. Age, hypertension, triglycerides, and antiplatelet use differed by conventional tests, and maximum pairwise SMDs indicated potentially important imbalance for age (0.54), female sex (0.45), hypertension (0.59), diabetes (0.46), waist circumference (0.44), diuretic use (0.44), and antiplatelet use (0.59) (Table 1). These differences support adjustment but also highlight the likelihood of residual confounding. Six-month blood-pressure data were available for 182 patients (76.5%): 45 receiving usual care, 48 with dietary management, 42 with omega-3 management, and 47 with combined management. Model-specific sample sizes were smaller when covariates were incomplete.

### 3.2. Population-Average Six-Month Cardiometabolic Change

Across the full cohort, blood pressure improved by the visit labeled three months and remained lower at the visit labeled six months. In adjusted GEE models, six-month SBP was 7.91 mmHg lower than the index value (95% CI −10.59 to −5.22; *p* < 0.001; standardized effect size, −0.42), and DBP was 4.75 mmHg lower (95% CI −6.27 to −3.23; *p* < 0.001; standardized effect size, −0.44). The adjusted HOMA-IR change corresponded to an approximate 9.9% reduction on the transformed scale (95% CI −15.3% to −4.1%; *p* = 0.001). These population-average changes describe follow-up trajectories and cannot be attributed to a specific recorded management category.

**Figure 2:**
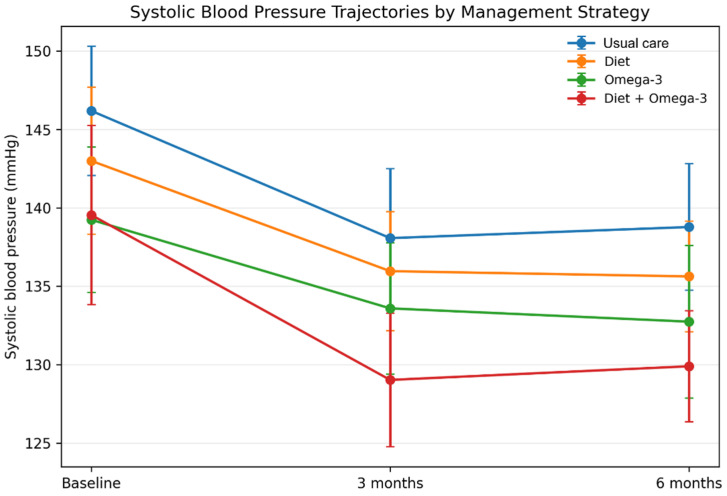
Unadjusted mean systolic blood-pressure trajectories by recorded management strategy. Error bars represent 95% confidence intervals. Adjusted omnibus group-by-time interaction: *p* = 0.987.

**Figure 3:**
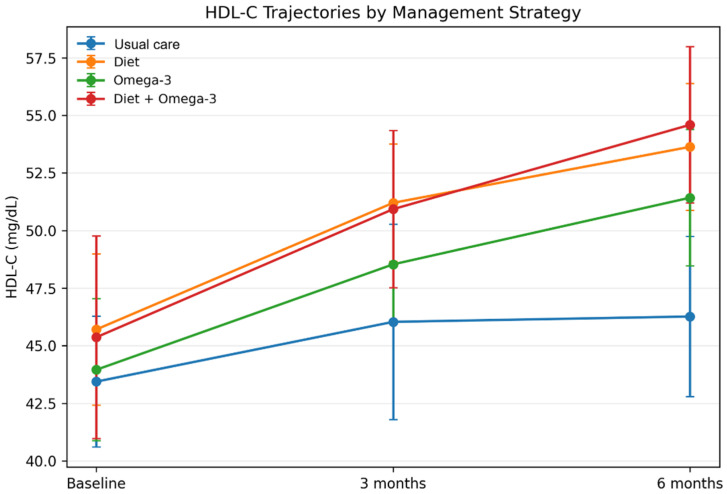
Unadjusted mean HDL-C trajectories by recorded management strategy. Error bars represent 95% confidence intervals. Adjusted omnibus group-by-time interaction: *p* = 0.179.

Total cholesterol declined by 15.32 mg/dL, triglycerides by 13.7%, and LDL-C by 12.41 mg/dL, whereas HDL-C increased by 7.16 mg/dL (all *p* < 0.001). Waist circumference decreased by 3.38 cm and legacy SCORE by 0.89 percentage points. IL-6 did not change significantly (−9.1%; 95% CI −23.7% to 8.3%; *p* = 0.285) (Table 2). Unadjusted SBP and HDL-C trajectories are shown in Figure 2 and Figure 3. Omnibus group-by-time interactions were nonsignificant for both parameters (*p* = 0.987 and *p* = 0.179, respectively).

### 3.3. Adjusted Comparisons of Management Strategies at Six Months

No omnibus group-by-time interaction was statistically significant for SBP, DBP, HOMA-IR, total cholesterol, triglycerides, HDL-C, LDL-C, waist circumference, IL-6, or legacy SCORE (all *p* ≥ 0.179), providing no evidence of differential longitudinal trajectories among the recorded management categories. The primary and additional adjusted comparisons are summarized separately in Table 3 and Table 4 to improve readability.

Several nominal six-month contrasts favored a recorded management category, including lower total cholesterol and LDL-C with dietary management, lower triglycerides with combined management, and lower carotid IMT in selected comparisons. These nominal findings did not survive correction for multiple testing and should not be interpreted as confirmatory treatment effects. The adjusted SBP difference for combined management versus usual care was −5.54 mmHg (95% CI −11.35 to 0.27; *p* = 0.062).

**Figure 4:**
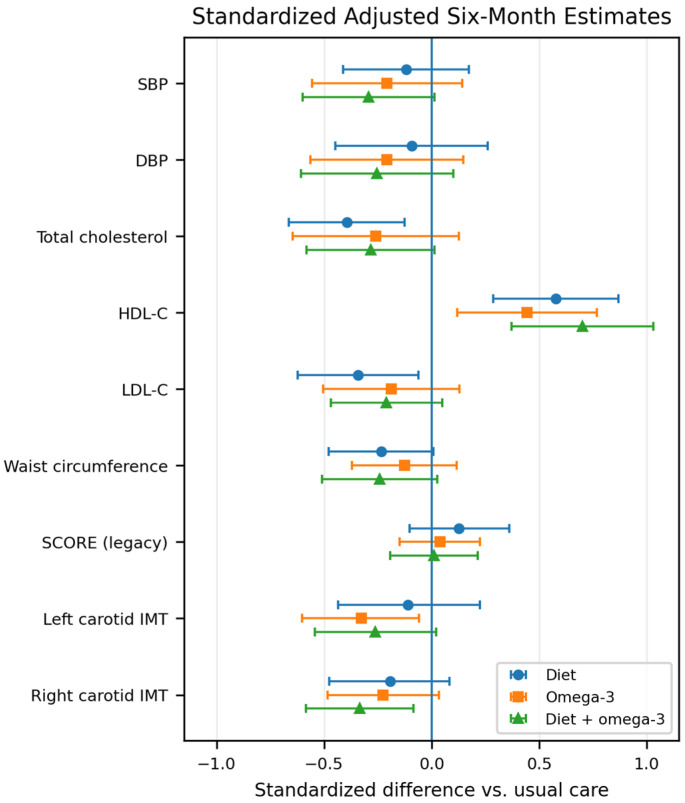
Forest plot of standardized adjusted six-month differences versus usual care for outcomes analyzed on their original scale. Positive values indicate a higher six-month outcome value than usual care and should not be interpreted uniformly as benefit because the favorable direction differs by outcome. HOMA-IR, triglycerides, and IL-6 are omitted because they were modeled on a log-transformed scale. Only the dietary-management and combined-management HDL-C associations survived false-discovery-rate correction; all other estimates are exploratory.

After Benjamini–Hochberg correction across 36 category-specific comparisons, only higher six-month HDL-C associated with dietary management versus usual care (adjusted difference +7.69 mg/dL; *q* = 0.0018; standardized effect size +0.58) and combined management versus usual care (+9.33 mg/dL; *q* = 0.0011; standardized effect size +0.70) remained statistically significant. No other between-category association survived false-discovery-rate correction. Because isolated HDL-C elevation is not an established causal surrogate for cardiovascular benefit [22], these findings remain exploratory. Figure 4 summarizes standardized adjusted estimates and confidence intervals for outcomes analyzed on their original scale.

### 3.4. Inflammatory and Vascular Outcomes

IL-6 showed substantial dispersion and nonuniform availability, particularly at the visit labeled three months. Neither the overall six-month IL-6 change nor any adjusted between-category association was statistically significant. These data do not support a consistent inflammatory association detectable through the single circulating marker available in this dataset.

At six months, lower left IMT in the omega-3 category and lower right IMT in the combined category were nominally significant, but neither association survived false-discovery-rate correction. Carotid IMT is a slowly changing structural measure, and reliable biological regression over six months would be difficult to distinguish from measurement variability, regression to the mean, or residual confounding. Moreover, a change in carotid IMT has not been established as a reliable surrogate for subsequent cardiovascular events [23]. Because study-specific intraobserver and interobserver reproducibility data were unavailable and no three-month IMT was measured, these findings should not be interpreted as vascular regression.

## 4. Discussion

### 4.1. Principal Findings

This retrospective longitudinal cohort yielded three principal observations. First, several blood-pressure, lipid, anthropometric, and insulin-resistance markers improved across the cohort during six months of follow-up. Second, no outcome demonstrated a statistically significant differential trajectory across the four recorded management categories. Third, after correction for 36 comparisons, only the dietary-management and combined-management associations with higher six-month HDL-C remained statistically significant. All other between-category findings were nonsignificant after false-discovery-rate correction.

The most defensible interpretation is that cardiometabolic recovery and clinical follow-up coincided with broad risk-factor improvement, rather than that a specific nutritional strategy produced these changes. Medication optimization, regression from acute or early post-acute abnormalities, spontaneous recovery, physical activity, rehabilitation, weight change, and other unmeasured behaviors may all have contributed. The isolated HDL-C signal should not be treated as a therapeutic endpoint. Mendelian-randomization evidence indicates that higher HDL-C concentration alone does not necessarily reduce myocardial-infarction risk [22], and the present study measured neither HDL function nor cardiovascular events.

### 4.2. Implications for Cardiometabolic Risk Reduction and Future Heart Failure Research

Hospitalization for COVID-19 may serve as a clinical opportunity to identify hypertension, obesity, diabetes, insulin resistance, dyslipidemia, and central adiposity that warrant evidence-based risk-factor management. These conditions are established cardiovascular risk factors and are also relevant to future HF risk [5,6]. Obesity is itself an established cardiovascular disease risk substrate [24]. However, this study did not adjudicate prior HF, assign HF stages, measure natriuretic peptides, assess left-ventricular structure or function, or evaluate filling pressures. References to HF and HF with preserved ejection fraction, therefore, provide a broader preventive context only and should not be interpreted as study outcomes or as evidence that the cohort had confirmed stage A HF.

The approximately 8 mmHg population-average reduction in SBP and the changes in waist circumference, lipids, and HOMA-IR may be clinically relevant risk-marker changes, but the study was not designed to connect them with incident HF or cardiovascular events. Future studies should combine standardized cardiometabolic interventions with natriuretic peptides, echocardiography, exercise capacity, patient-reported outcomes, and adjudicated cardiovascular and HF events.

A pragmatic post-discharge pathway could reassess blood pressure, glycemic control, body composition, lipids, physical activity, medications, and cardiovascular symptoms. Patients with dyspnea, chest pain, palpitations, syncope, exercise intolerance, abnormal biomarkers, or abnormal electrocardiographic findings require symptom-directed evaluation consistent with expert guidance [4]. The current results support structured reassessment of modifiable risk factors, not a specific claim of HF prevention.

### 4.3. Dietary and Omega-3 Management: Evidence and Interpretation

Dietary patterns emphasizing vegetables, fruit, whole grains, legumes, nuts, fish, and unsaturated fats while limiting sodium, refined carbohydrates, and ultra-processed foods are established components of cardiovascular prevention [7,8]. Secondary analyses of Mediterranean-diet trials have also reported favorable effects on HF-related biomarkers and incident HF risk [25,26]. In the present study, however, dietary management was a heterogeneous documentation category rather than a standardized intervention. Counseling content, intensity, reinforcement, adherence, and concurrent behavioral changes were incompletely captured. The observed estimates, therefore, cannot be compared directly with effects from protocolized dietary trials.

Omega-3 supplementation likewise has formulation- and dose-dependent effects [9,10,11,27]. The source records did not permit consistent identification of product, EPA/DHA dose, duration, continuation, or adherence. The nominal triglyceride and HDL-C findings, therefore, do not establish a pharmacological effect of omega-3. A future trial would require a specified formulation and dose, objective adherence assessment, predefined cointerventions, and a contemporaneous control group.

Confounding by indication remains a central limitation. Patients receiving dietary counseling or omega-3 recommendations may have differed in motivation, clinician contact, baseline risk, access to care, or likelihood of receiving other treatments. Baseline SMDs demonstrate substantial imbalance, and multivariable adjustment cannot remove bias from unmeasured factors. Propensity-score matching or weighting would not solve this problem because key treatment-selection variables were unavailable and the four-group sample was modest; matching could discard participants, while weighting could produce unstable estimates if overlaps were poor [19]. The results should therefore be viewed as hypothesis-generating associations.

### 4.4. Interpretation of Inflammatory and Vascular Findings 

Persistent immune dysregulation and metabolic abnormalities have been implicated in post-acute COVID-19 [28,29,30], while acute infection may worsen insulin resistance and glycometabolic control [31,32]. IL-6 did not improve consistently, potentially because of biological heterogeneity, assay timing, nonuniform availability, intermittent inflammatory activity, and the limited sensitivity of a single cytokine as a recovery marker.

Recovery after SARS-CoV-2 infection involves multiple physiological systems. Previous work has described changes in blood pressure, body composition, bone health, physical performance, cognition, and mental health [33,34,35,36]. Accordingly, the cardiometabolic trajectories observed here may reflect interactions among residual inflammation, deconditioning, muscle loss, spontaneous recovery, physical activity, rehabilitation, diet, medication changes, and social circumstances rather than an isolated nutritional effect. Longitudinal evidence also suggests that COVID-19-associated vascular dysfunction may partially recover over time [37]. A Romanian cohort further found that NT-proBNP, troponin I, and IL-6 identified a high-risk acute pulmonary-edema phenotype associated with mortality and persistent pulmonary sequelae [38].

The carotid IMT findings require particular caution. A structural arterial measure generally changes slowly, and an apparent six-month difference of several hundredths of a millimeter may be within the range influenced by acquisition, reader, and positioning variability. The Mannheim protocol was followed [17], but study-specific intraobserver and interobserver coefficients were not recorded. The nominal associations did not survive multiplicity correction, and evidence does not support individual short-term IMT progression as a validated surrogate for cardiovascular events [23]. No claim of structural regression is therefore warranted.

### 4.5. Clinical Interpretation and Future Study Design

The broad cohort-level improvement suggests that post-hospitalization follow-up may be a useful setting for medication reconciliation and risk-factor reassessment. It does not identify which component of care produced the changes. A definitive study should use standardized dietary and omega-3 protocols; record adherence, physical activity, rehabilitation, socioeconomic status, and weight-loss behaviors; capture acute severity, vaccination, viral period, and acute treatment; and collect time-updated cardiometabolic medications, including statins, ezetimibe, PCSK9 inhibitors, GLP-1 receptor agonists, SGLT2 inhibitors, antihypertensive agents, and anti-obesity therapies.

Prospective work should also prespecify a primary endpoint and sample-size calculation, assess treatment overlap before causal modeling, and consider randomized allocation when feasible. Repeated cardiac biomarkers, echocardiography, vascular imaging with blinded reproducibility assessment, functional capacity, and patient-reported outcomes would permit clinically meaningful interpretation. HF-specific instruments should be reserved for cohorts with established or suspected HF.

### 4.6. Strengths and Limitations

Strengths include the six-month longitudinal design, simultaneous evaluation of hemodynamic, metabolic, inflammatory, and vascular domains, transparent outcome-specific denominators, repeated-measures modeling, baseline-adjusted comparisons, robust standard errors, baseline SMD reporting, and false-discovery-rate correction. The study also illustrates the limits of drawing causal conclusions from heterogeneous management categories abstracted from routine care.

This study has several major limitations. First, its retrospective, single-center, nonrandomized design is vulnerable to confounding by indication and residual confounding. Baseline imbalances were substantial, and the determinants of treatment selection were incompletely measured. Propensity matching or inverse-probability weighting was not undertaken because of the four-group design, modest sample, uncertain overlap, and missing key treatment-selection variables; such methods would not correct unmeasured confounding. Second, dietary and omega-3 categories were derived from heterogeneous free-text and checkbox documentation. Product, dose, duration, counseling intensity, continuation, and adherence were not standardized, creating exposure misclassification and information bias. Third, changes in pharmacological therapy were not recorded. Initiation or intensification of statins, ezetimibe, PCSK9 inhibitors, GLP-1 receptor agonists, SGLT2 inhibitors, antihypertensive agents, or weight-loss therapy could plausibly explain part of the observed changes.

Fourth, the cohort spanned 2020–2024, including ancestral, Alpha, Delta, and Omicron-dominant periods, evolving vaccination uptake, changing admission thresholds, and major changes in acute treatment, including corticosteroid-based care [39,40]. Viral variant, vaccination status, acute treatment, and standardized severity indices were unavailable, so recovery trajectories may reflect pandemic phase as well as patient-level severity. Fifth, physical activity, rehabilitation participation, dietary changes outside recorded counseling, socioeconomic circumstances, and other behaviors were not measured. Sixth, no formal sample-size calculation was performed, and incomplete outcome-specific follow-up further reduced precision; negative findings may therefore reflect type II error and should be interpreted with confidence intervals. Seventh, exact follow-up dates were not retained, the baseline window extended to 30 days after discharge, and the visits labeled three and six months may have varied in timing. Eighth, missingness may have been informative, and no imputation was performed. Ninth, the number of blood-pressure readings was not recorded consistently. Tenth, no HF-specific biomarkers, cardiac imaging, clinical HF outcomes, or cardiovascular events were available, precluding HF staging or claims of HF prevention. Eleventh, legacy SCORE was retained only because it was stored in the database; it is not a contemporary individualized risk estimate, particularly in diabetes, and SCORE2/SCORE2-Diabetes could not be reconstructed uniformly [15,16]. Twelfth, no study-specific IMT reproducibility coefficients were available, the six-month interval is short for structural arterial change, and nominal IMT associations did not survive multiplicity correction. All comparative and vascular findings remain exploratory.

## 5. Conclusions

In this single-center retrospective cohort, several cardiometabolic risk markers improved over six months after COVID-19 hospitalization. However, no recorded management category showed a significantly different longitudinal trajectory, and only the dietary-management and combined-management associations with higher HDL-C remained statistically significant after false-discovery-rate correction. Isolated HDL-C elevation does not establish cardiovascular benefit. Because management exposures were heterogeneous and nonrandomized, adherence and medication changes were unavailable, pandemic phase and acute severity were not controlled, and HF-specific measures and clinical events were not assessed, the findings do not demonstrate efficacy of dietary counseling or omega-3 supplementation and do not demonstrate prevention of HF. Prospective, adequately powered, protocolized studies are required.

## Figures and Tables

**Table 1:** Baseline characteristics according to recorded management strategy.

Characteristic	Usual Care (*n =* 64)	Diet (*n =* 60)	Omega-3 (*n =* 59)	Diet + Omega-3 (*n =* 55)	*p* Value	Max SMD
Age, years	57.4 ± 11.5	54.8 ± 9.2	51.5 ± 10.3	51.8 ± 10.4	0.005	0.54
Female sex	43 (67.2)	31 (51.7)	31 (52.5)	25 (45.5)	0.100	0.45
BMI, kg/m^2^	33.9 ± 4.9	34.5 ± 4.8	33.0 ± 4.2	33.4 ± 4.3	0.329	0.33
Hypertension	57 (89.1)	55 (91.7)	46 (78.0)	38 (69.1)	0.005	0.59
Diabetes mellitus	34 (53.1)	24 (40.0)	22 (37.3)	17 (30.9)	0.087	0.46
Current smoking	9 (14.1)	11 (18.3)	14 (23.7)	13 (23.6)	0.476	0.25
Systolic blood pressure, mmHg	146.2 ± 16.7	143.0 ± 18.6	139.2 ± 18.2	139.5 ± 21.7	0.141	0.40
Diastolic blood pressure, mmHg	84.9 ± 9.9	83.3 ± 9.8	82.4 ± 10.9	83.6 ± 12.4	0.625	0.24
HOMA-IR	3.34 [1.84–4.99]	3.50 [2.65–5.32]	3.81 [2.68–5.58]	3.85 [2.16–6.50]	0.512	—
Total cholesterol, mg/dL	215.5 ± 53.3	217.1 ± 40.8	229.3 ± 53.9	225.4 ± 51.6	0.374	0.26
Triglycerides, mg/dL	155.0 [120.0–191.0]	149.3 [112.3–206.0]	195.0 [149.5–269.8]	196.0 [129.2–244.0]	0.002	—
HDL-C, mg/dL	43.4 ± 11.5	45.7 ± 13.0	44.0 ± 12.1	45.4 ± 16.6	0.752	0.19
LDL-C, mg/dL	154.2 ± 53.9	149.4 ± 44.9	154.2 ± 60.5	148.9 ± 50.8	0.913	0.10
Waist circumference, cm	109.4 ± 11.8	111.3 ± 10.9	107.1 ± 8.1	109.1 ± 10.8	0.190	0.44
IL-6, pg/mL	5.22 [1.04–16.81]	3.71 [0.00–9.30]	3.94 [0.00–8.88]	1.39 [0.00–8.70]	0.115	—
SCORE (legacy), %	3.0 [1.0–5.0]	3.0 [1.0–5.0]	2.0 [1.0–4.0]	2.0 [1.0–5.0]	0.594	—
Left carotid IMT, mm	0.619 ± 0.127	0.606 ± 0.121	0.633 ± 0.166	0.596 ± 0.135	0.518	0.24
Right carotid IMT, mm	0.601 ± 0.099	0.589 ± 0.117	0.587 ± 0.142	0.588 ± 0.121	0.916	0.12
Beta-blocker use	26 (40.6)	21 (35.0)	22 (37.3)	18 (32.7)	0.830	0.16
ACE inhibitor or ARB use	45 (70.3)	41 (68.3)	34 (57.6)	29 (52.7)	0.146	0.37
Diuretic use	21 (32.8)	25 (41.7)	19 (32.2)	12 (21.8)	0.159	0.44
Statin use	11 (17.2)	10 (16.7)	11 (18.6)	7 (12.7)	0.852	0.16
Antiplatelet use	34 (53.1)	23 (38.3)	22 (37.3)	14 (25.5)	0.021	0.59

Data are mean ± standard deviation, median [interquartile range], or *n* (%). *p* values were obtained using one-way ANOVA, Kruskal–Wallis, or chi-squared tests. Max SMD denotes the maximum absolute pairwise standardized mean difference across the four management groups, provided as a scale-free descriptor of baseline balance; values below 0.10 indicate negligible imbalance and values of about 0.20 or above indicate potentially meaningful imbalance. SMD is not reported (—) for variables summarized as medians with interquartile ranges, for which a standardized mean difference is not appropriate. ACE, angiotensin-converting enzyme; ARB, angiotensin-receptor blocker; BMI, body mass index; HDL-C, high-density lipoprotein cholesterol; HOMA-IR, homeostasis model assessment of insulin resistance; IL-6, interleukin-6; IMT, intima-media thickness; LDL-C, low-density lipoprotein cholesterol; SCORE, Systematic Coronary Risk Evaluation.

**Table 2:** Longitudinal cardiometabolic outcomes and adjusted six-month differences versus the index assessment.

Outcome	Index Assessment	3 Months	6 Months	Adjusted Difference at 6 Months vs. Index (95% CI)	*p*
Systolic blood pressure, mmHg	142.1 ± 18.9 (*n =* 237)	134.2 ± 14.8 (*n* = 188)	134.3 ± 14.0 (*n* = 182)	−7.91 (−10.59 to −5.22)	<0.001
Diastolic blood pressure, mmHg	83.6 ± 10.7 (*n* = 237)	78.5 ± 9.3 (*n* = 188)	78.4 ± 9.2 (*n* = 182)	−4.75 (−6.27 to −3.23)	<0.001
HOMA-IR	3.55 [2.42–5.52] (*n* = 234)	3.68 [2.47–5.40] (*n* = 179)	3.29 [2.43–4.71] (*n* = 173)	−9.9% (−15.3% to −4.1%)	0.001
Total cholesterol, mg/dL	221.7 ± 50.2 (*n* = 237)	209.8 ± 46.0 (*n* = 188)	207.0 ± 45.5 (*n* = 182)	−15.32 (−22.01 to −8.63)	<0.001
Triglycerides, mg/dL	171.0 [128.0–228.1] (*n* = 237)	143.4 [108.6–197.2] (*n* = 188)	138.2 [108.4–187.2] (*n* = 182)	−13.7% (−18.6% to −8.5%)	<0.001
HDL-C, mg/dL	44.6 ± 13.3 (*n* = 237)	49.3 ± 11.3 (*n* = 188)	51.6 ± 11.3 (*n* = 182)	7.16 (5.31 to 9.00)	<0.001
LDL-C, mg/dL	151.8 ± 52.5 (*n* = 227)	145.9 ± 44.6 (*n* = 184)	143.6 ± 40.4 (*n* = 177)	−12.41 (−18.88 to −5.95)	<0.001
Waist circumference, cm	109.2 ± 10.6 (n = 237)	107.0 ± 10.7 (*n* = 185)	105.7 ± 10.8 (*n* = 180)	−3.38 (−4.24 to −2.51)	<0.001
IL-6, pg/mL	4.30 [0.00–9.81] (*n* = 200)	3.00 [0.00–20.52] (*n* = 88)	4.65 [0.22–8.30] (*n* = 164)	−9.1% (−23.7% to 8.3%)	0.285
SCORE (legacy), %	3.78 ± 4.16 (*n* = 230)	3.01 ± 3.06 (*n* = 186)	3.03 ± 3.03 (*n* = 179)	−0.89 (−1.28 to −0.49)	<0.001

Values are mean ± standard deviation or median [interquartile range]. Adjusted differences were obtained from GEE models, including time, management group, age, sex, and baseline BMI. HOMA-IR, triglycerides, and IL-6 were modeled as log(1 + value) and are shown as approximate percentage differences. Standardized effect sizes reported in the text were calculated by dividing the adjusted six-month change by the corresponding overall baseline standard deviation. CI, confidence interval; GEE, generalized estimating equation.

**Table 3:** Primary adjusted six-month comparisons versus usual care.

Outcome (Model *n*)	Diet vs. Usual Care	Omega-3 vs. Usual Care	Combined vs. Usual Care	GEE Interaction *p*
SBP, mmHg (*n* = 180)	−2.24 (−7.77 to 3.29); *p* = 0.427	−3.91 (−10.52 to 2.70); *p* = 0.246	−5.54 (−11.35 to 0.27); *p* = 0.062	0.987
DBP, mmHg (*n* = 180)	−0.99 (−4.78 to 2.79); *p* = 0.607	−2.23 (−6.03 to 1.57); *p* = 0.250	−2.71 (−6.50 to 1.08); *p* = 0.161	0.850
Total cholesterol, mg/dL (*n* = 180)	−19.81 (−33.37 to −6.25); *p* = 0.004	−13.00 (−32.38 to 6.37); *p* = 0.188	−14.25 (−29.15 to 0.65); *p* = 0.061	0.386
HDL-C, mg/dL (*n* = 180)	+7.69 (3.81 to 11.56); *p* < 0.001	+5.91 (1.59 to 10.23); *p* = 0.007	+9.33 (4.93 to 13.72); *p* < 0.001	0.179
LDL-C, mg/dL (*n* = 168)	−17.91 (−32.63 to −3.19); *p* = 0.017	−9.80 (−26.44 to 6.84); *p* = 0.248	−10.99 (−24.56 to 2.57); *p* = 0.112	0.873
Waist circumference, cm (*n* = 178)	−2.49 (−5.07 to 0.09); *p* = 0.059	−1.34 (−3.92 to 1.25); *p* = 0.311	−2.55 (−5.39 to 0.29); *p* = 0.079	0.364

Values are adjusted differences (95% CI); nominal *p* value. Models included the baseline outcome, age, sex, baseline BMI, hypertension, diabetes, and current smoking, with relevant baseline therapy added by outcome. HC3 robust standard errors were used. Only the dietary-management and combined-management HDL-C associations survived false-discovery-rate correction. Interaction *p* values test differential trajectories across three visits.

**Table 4:** Additional exploratory adjusted six-month comparisons versus usual care.

Outcome (Model *n*)	Diet vs. Usual Care	Omega-3 vs. Usual Care	Combined vs. Usual Care	GEE Interaction *p*
HOMA-IR, % (*n* = 171)	−6.2% (−19.4 to 9.1); *p* = 0.407	+1.7% (−14.6 to 21.1); *p* = 0.847	−3.1% (−17.5 to 13.8); *p* = 0.703	0.437
Triglycerides, % (*n* = 180)	−11.1% (−22.3 to 1.7); *p* = 0.087	−8.9% (−22.2 to 6.6); *p* = 0.243	−16.3% (−27.4 to −3.4); *p* = 0.015	0.183
IL-6, % (*n* = 148)	−5.5% (−38.1 to 44.2); *p* = 0.793	−22.7% (−50.1 to 19.8); *p* = 0.249	+2.0% (−30.6 to 49.9); *p* = 0.919	0.565
SCORE, percentage points (*n* = 172)	+0.53 (−0.43 to 1.50); *p* = 0.277	+0.16 (−0.62 to 0.94); *p* = 0.685	+0.05 (−0.80 to 0.89); *p* = 0.916	0.977
Left IMT, mm (*n* = 177)	−0.015 (−0.060 to 0.031); *p* = 0.521	−0.045 (−0.083 to −0.008); *p* = 0.017	−0.036 (−0.075 to 0.003); *p* = 0.072	Not available
Right IMT, mm (*n* = 177)	−0.023 (−0.057 to 0.010); *p* = 0.172	−0.027 (−0.058 to 0.004); *p* = 0.091	−0.040 (−0.070 to −0.010); *p* = 0.010	Not available

Values are adjusted differences (95% CI); nominal *p* value. HOMA-IR, triglycerides, and IL-6 were modeled as log(1 + value) and are shown as approximate percentage differences. No three-month IMT measurements were available, so trajectory interaction tests could not be calculated. None of the associations in this table survived false-discovery-rate correction.

## Data Availability

The de-identified data supporting the findings of this study are not publicly available because of participant confidentiality and institutional data-protection requirements. Data may be made available by the corresponding authors upon reasonable request and subject to institutional approval.

## Abbreviations

The following abbreviations are used in this manuscript:

ACE	angiotensin-converting enzyme
ANCOVA	analysis of covariance
ARB	angiotensin-receptor blocker
BMI	body mass index
CI	confidence interval
COVID-19	coronavirus disease 2019
CV	coefficient of variation
DBP	diastolic blood pressure
DHA	docosahexaenoic acid
ECLIA	electrochemiluminescence immunoassay
EPA	eicosapentaenoic acid
GEE	generalized estimating equation
HDL-C	high-density lipoprotein cholesterol
HF	heart failure
HFpEF	heart failure with preserved ejection fraction
HOMA-IR	homeostasis model assessment of insulin resistance
IL-6	interleukin-6
IMT	intima-media thickness
LDL-C	low-density lipoprotein cholesterol
NT-proBNP	N-terminal pro-B-type natriuretic peptide
RT-PCR	reverse transcription-polymerase chain reaction
SBP	systolic blood pressure
SCORE	Systematic Coronary Risk Evaluation
SMD	standardized mean difference
STROBE	Strengthening the Reporting of Observational Studies in Epidemiology
WHO	World Health Organization
